# Anesthetic Management of Stiff-Person Syndrome During Prolonged Lateral Skull Base Surgery: A Case Report and Phenotype-Informed Review

**DOI:** 10.3390/jcm15218217

**Published:** 2026-10-22

**Authors:** Jianhao Zhang, Haisong Lu, Yang Zhao, Guodong Feng, Yuguan Zhang

**Affiliations:** 1 Department of Anesthesiology, Peking Union Medical College Hospital, Chinese Academy of Medical Sciences & Peking Union Medical College, Beijing 100730, China; zhangjh20001029@163.com (J.Z.); luhaisong@aliyun.com (H.L.); 2 Department of Anesthesiology, Plastic Surgery Hospital, Chinese Academy of Medical Sciences & Peking Union Medical College, Beijing 100144, China; 3 Department of Otolaryngology–Head and Neck Surgery, Peking Union Medical College Hospital, Chinese Academy of Medical Sciences & Peking Union Medical College, Beijing 100730, China; zhaoyang11889@126.com (Y.Z.); fgdent@163.com (G.F.)

**Keywords:** stiff-person syndrome, total intravenous anesthesia, neuromuscular monitoring, lateral skull base surgery, case report

## Abstract

**Background:** Stiff-person syndrome (SPS) is a rare autoimmune neurological disorder characterized by progressive rigidity and episodic spasms. Perioperative evidence consists mainly of case reports, underscoring the importance of incorporating heterogeneity across the stiff-person spectrum disorder (SPSD) into perioperative assessment. To our knowledge, this is the first published report of SPS management during prolonged lateral skull base surgery. **Case Presentation:** A 56-year-old man with anti-amphiphysin antibody-positive, lower-limb-predominant SPSD without documented generalized spasms and a skull base phosphaturic mesenchymal tumor underwent an 11 h radical resection. Total intravenous anesthesia with propofol, remifentanil, and dexmedetomidine was used without neuromuscular blocking agents because facial and lower cranial nerve monitoring was required. Bispectral index values were maintained at 30–50, and serial quantitative train-of-four monitoring showed four responses with ratios of 100–125% throughout the procedure. No intraoperative spasm, movement, or hemodynamic instability occurred. The patient was extubated 3.5 h after intensive care unit admission, and serum phosphorus normalized within 48 h. A focused review of published reports identified no previous lateral skull base surgery in SPS and limited prolonged quantitative neuromuscular monitoring data. **Conclusions:** Prolonged muscle-relaxant-free total intravenous anesthesia was feasible in this selected SPS phenotype. Perioperative planning should integrate individual spasm history, bulbar or respiratory involvement, medication burden, surgical requirements, and SPSD phenotype. The proposed phenotype-informed framework organizes these considerations for individualized perioperative planning and provides a basis for future evaluation.

## 1. Introduction

Stiff-person syndrome (SPS) is a rare autoimmune neurological disorder with an estimated prevalence of 1–2 per million, characterized by progressive muscle rigidity and episodic painful spasms [1,2]. The underlying pathology involves impaired central γ-aminobutyric acid (GABA)-ergic inhibitory neurotransmission. Approximately 60–80% of patients harbor serum antibodies against glutamic acid decarboxylase 65 (GAD65), the rate-limiting enzyme for GABA synthesis [1,2,3]. Other autoantibodies, including anti-amphiphysin and anti-glycine receptor (GlyR) antibodies, are associated with distinct SPS subtypes [4,5].

Martinez-Hernandez et al. (2016) classified SPS into four clinical types in a cohort of 121 patients: classic SPS, stiff-limb syndrome (SLS), SPS-plus/progressive encephalomyelitis with rigidity and myoclonus (PERM), and overlapping syndromes, formally establishing the “stiff-person spectrum disorder” (SPSD) concept [4]. This classification was further elaborated by Termsarasab et al. [1] and in Dalakas’s comprehensive review [2]. Clinical heterogeneity across SPSD subtypes, including antibody profiles, spasm frequency, trigger characteristics, and prognosis, may inform perioperative assessment and warrants systematic evaluation in anesthesia studies.

The perioperative management of SPS presents unique challenges. Patients chronically receive benzodiazepines (GABA-A agonists) and baclofen (GABA-B agonist) to compensate for endogenous GABA deficiency, while most general anesthetics also act on GABA receptors [6,7,8]. Since the seminal report by Johnson and Miller in 1995 [6], published reports have described SPS anesthesia in thymectomy [9,10,11], orthopedic [12,13,14], obstetric [15], abdominal [7,16,17,18,19], and other procedures. These reports contain conflicting conclusions regarding neuromuscular blocking agents (NMBAs) and the safety of volatile anesthetics [6,20,21]. Sidransky et al. (2013) provided a case-based discussion of anesthetic considerations in SPS [22]. Our focused review identified limited reports addressing lateral skull base surgery and phenotype-informed perioperative assessment.

To our knowledge, we report the first published case of lateral skull base surgery in an SPS patient and, through a focused review of published reports, propose phenotype-informed updates to SPS anesthetic management.

This report was prepared in accordance with the CARE guideline.

## 2. Case Presentation

### 2.1. Patient Information, Diagnostic Assessment, and Clinical Timeline

A 56-year-old male (weight 76 kg) presented with progressive lower limb rigidity and pain over approximately two years. Symptoms were triggered by postural changes (standing, walking) and relieved in the supine position. No generalized episodic spasms were documented throughout the disease course. Physical examination revealed reduced tendon reflexes and impaired pinprick and vibration sensation in both lower extremities. The completed CARE checklist is provided as Checklist S1.

Serum anti-amphiphysin antibody was positive (titer 1:100), with negative anti-GAD65 and anti-GlyR antibodies. Electromyography demonstrated abundant continuous motor unit action potential (MUAP) discharges in the left tibialis anterior muscle at rest, markedly increased by standing and abolished by intravenous diazepam 5 mg, providing electrophysiological support for SPSD [1,2,23]. The patient had previously responded to intravenous immunoglobulin combined with methylprednisolone pulse therapy. A trial prednisone taper to 15 mg/day resulted in dysphagia, which resolved upon dose increase to 25 mg/day. Preoperative medications: prednisone 25 mg qd, baclofen 10 mg tid, clonazepam 1 mg bid, oxcarbazepine 0.3 g bid, diclofenac 100 mg qd. Comorbidities included hypertension and type 2 diabetes.

Whole-body FDG PET/CT revealed a hypermetabolic mass in the right jugular foramen region (SUVmax 12.9) with adjacent bone destruction. Concurrent laboratory tests showed severe hypophosphatemia (serum phosphorus 0.31–0.50 mmol/L), elevated alkaline phosphatase (262–357 U/L), bilateral femoral neck fractures, multiple rib fractures, and T4–L1 vertebral compression. These findings were consistent with tumor-induced osteomalacia (TIO). A first-stage biopsy under monitored anesthesia care (MAC) with dexmedetomidine (Yangtze River Pharmaceutical Group Co., Ltd., Taizhou, China) sedation was well tolerated. Pathology confirmed a phosphaturic mesenchymal tumor (PMT). Preoperative phosphorus supplementation with neutral phosphate solution, calcitriol, and vitamin D was initiated. The clinical presentation reflected two potentially overlapping processes. TIO commonly causes bone pain, muscle weakness, impaired mobility, and fragility fractures [24] and could therefore account for part of the lower-limb pain and functional limitation. In contrast, persistent rigidity, continuous motor-unit activity at rest that increased with standing and was abolished by diazepam, anti-amphiphysin seropositivity, and the prior response to immunotherapy provided convergent support for lower-limb-predominant SPSD [23,25]. Pathological confirmation of PMT together with severe hypophosphatemia, elevated alkaline phosphatase, and multiple fractures strongly supported concurrent PMT-associated TIO. Serum phosphorus normalized within 48 h after resection in the setting of phosphate, calcitriol, and vitamin D therapy.

**Figure 1:**
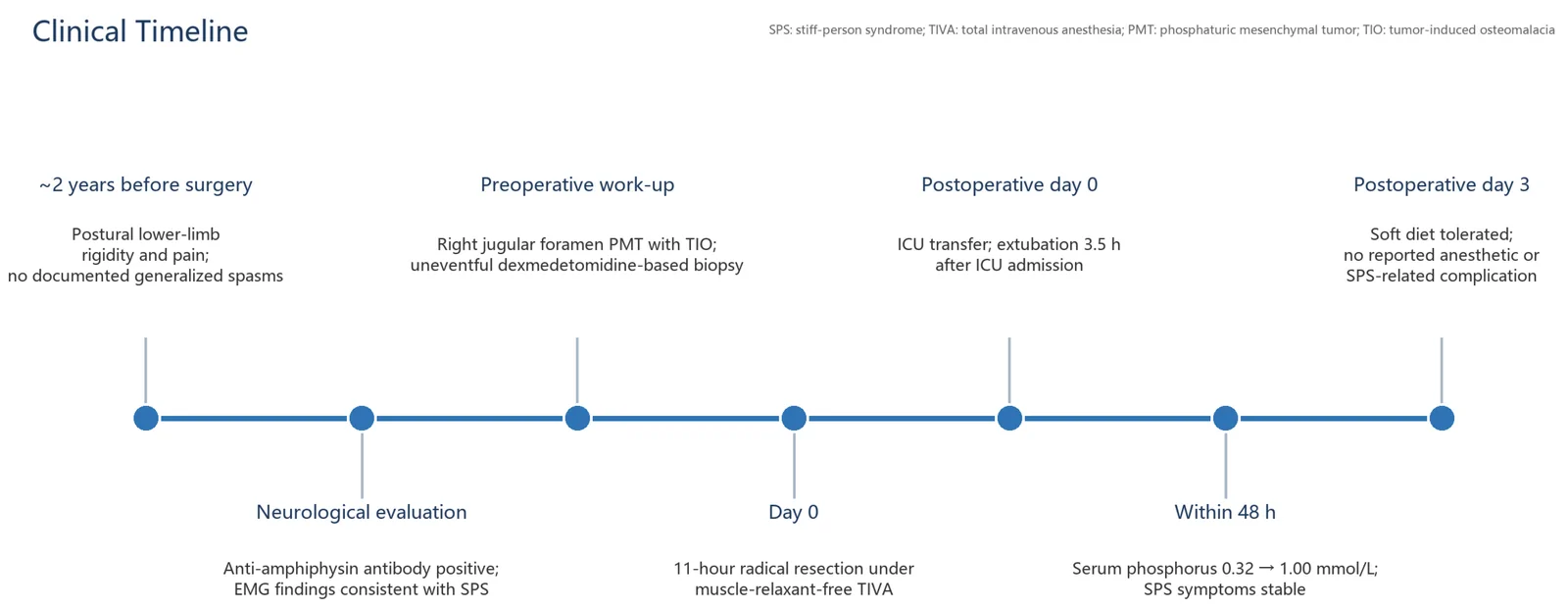
Clinical timeline of the patient’s symptoms, diagnostic work-up, surgery, and early postoperative course.

The patient subsequently underwent elective radical resection of the PMT via a right infratemporal fossa approach, with concurrent great auricular nerve-to-facial nerve grafting and tympanic cavity reconstruction using sternocleidomastoid and temporalis muscle flaps. Intraoperative facial and lower cranial nerve monitoring necessitated avoidance of NMBAs. The operation lasted 11 h. The clinical course is summarized in Figure 1.

### 2.2. Perioperative Anesthetic Management

On the morning of surgery, he received his usual prednisone 25 mg, baclofen 10 mg, clonazepam 1 mg, oxcarbazepine 300 mg, and diclofenac sodium 100 mg. No separate stress-dose glucocorticoid regimen was used; dexamethasone (Tianjin Pharma Heping (Tianjin) Pharmaceutical Co., Ltd., Tianjin, China) 5 mg was administered intraoperatively for postoperative nausea and vomiting prophylaxis. Because preoperative high-sensitivity cardiac troponin I (hs-cTnI) was mildly elevated (128 ng/L), bedside transthoracic echocardiography was performed before induction and showed normal cardiac structure and function. Anesthesia was induced with propofol (Diprivan; Corden Pharma S.p.A., Caponago, Italy) delivered by effect-site target-controlled infusion (TCI) at 3 μg/mL using a TCI pump (Mindray, Shenzhen, China), midazolam (Liyuexi; Jiangsu Nhwa Pharmaceutical Co., Ltd., Xuzhou, China) 2 mg, and sufentanil (Yichang Humanwell Pharmaceutical Co., Ltd., Yichang, China) 0.25 μg/kg administered slowly. Parecoxib (Dynastat; Pharmacia & Upjohn Company LLC, Kalamazoo, MI, USA) 40 mg was given for multimodal preemptive analgesia. Video laryngoscopy using a video laryngoscope (Zhejiang UE Medical Corp., Xianju, China) showed a modified Cormack-Lehane grade 1 view. After topical lidocaine (China Otsuka Pharmaceutical Co., Ltd., Tianjin, China) anesthesia, tracheal intubation was performed without NMBAs. Anesthesia was maintained with propofol TCI at 2–2.5 μg/mL, remifentanil (Yichang Humanwell Pharmaceutical Co., Ltd., Yichang, China) 0.15 μg/kg/min, and dexmedetomidine (loading dose 1 μg/kg; maintenance 0.3–0.4 μg/kg/h). Because microsurgical dissection occurred near critical neurovascular structures, BIS, measured with a BIS monitor (Covidien LLC, Boulder, CO, USA), was maintained at 30–50 to titrate anesthetic depth and minimize movement. Additional sufentanil was administered as needed, and blood pressure was maintained within ±20% of baseline.

Monitoring included five-lead ECG, invasive arterial pressure, BIS, bladder temperature, and serial acceleromyographic quantitative TOF monitoring (Mindray, Shenzhen, China; ulnar nerve/adductor pollicis). TOF count remained consistently at 4 with TOF ratio 100–125% throughout the 11-h procedure, and no fade was observed. No patient movement or spasm occurred intraoperatively. Total crystalloid input was 7300 mL, estimated blood loss 300–400 mL (no transfusion), and urine output 4400 mL.

Considering the risk of upper airway edema following prolonged lateral skull base surgery, potential GABAergic drug accumulation leading to delayed emergence [6,7,8], and published precedents of inadequate respiration despite normalized TOF [7,11,26], a strategy of delayed extubation was adopted. The patient was transferred to the ICU with the endotracheal tube in situ. Extubation was performed 3.5 h after ICU admission upon confirmation of spontaneous tidal volume ≥8 mL/kg, sustained head lift >5 s, and strong hand grip. Postoperative analgesia was provided via patient-controlled intravenous analgesia combined with NSAIDs.

### 2.3. Outcome and Follow-Up

During the available inpatient follow-up, the postoperative course was uneventful. SPS symptoms remained stable, with no change in baclofen or clonazepam dosing. Serum phosphorus normalized within 48 h (0.32 → 1.00 mmol/L), and hs-cTnI remained below the preoperative value (128 → 83 → 94 ng/L). He tolerated a soft diet by postoperative day 3.

## 3. Discussion

### 3.1. Anesthetic Management

SPS patients rely on chronic benzodiazepine and baclofen therapy to compensate for endogenous GABA deficiency. Since most general anesthetics also potentiate GABAergic signaling, the combined effect may lead to postoperative hypotonia and delayed emergence. This concern was recognized early in the SPS anesthesia literature. Johnson and Miller (1995) reported that a patient on diazepam 100 mg/day who received vecuronium under isoflurane required overnight mechanical ventilation despite full TOF recovery and neostigmine reversal [6]. Murphy and Shorten (2000) reported 57% TOF fade during sevoflurane induction in a patient with stiff-baby syndrome who had received no NMBA [26]. Bouw et al. [7] subsequently described a baclofen-treated SPS patient who exhibited clinical weakness with a tidal volume of only 200 mL despite normal TOF after isoflurane anesthesia with atracurium. Pharmacokinetic simulation excluded NMBA and opioid residuals, and the authors attributed the weakness to synergistic baclofen and isoflurane action at GABA-B receptors. This proposed interaction has subsequently been discussed in case reports and reviews [8,12,16].

The extent of additive GABAergic effects may vary with SPS severity, the preoperative GABAergic medication burden, and the duration of surgery [6,8,16]. In this case, the daily clonazepam dose (2 mg/day) differed substantially from the diazepam regimen in Johnson’s report (100 mg/day), although direct dose equivalence between benzodiazepines is imprecise. The absence of respiratory muscle involvement and avoidance of volatile anesthetics may also have contributed to the uncomplicated postoperative recovery.

Whether to use NMBAs remains a central point of controversy in the literature. Several groups have reported safe NMBA use in SPS patients. Obara et al. administered vecuronium without complications across three anesthetics in the same patient [19], Qin et al. observed normal pharmacodynamics with cumulative rocuronium 90 mg [10], and Cassavaugh and Oravitz documented uneventful use of both succinylcholine and rocuronium across three separate procedures [17]. Matsubara et al. (2015) described uneventful anesthetic management for an orthopedic procedure using rocuronium and, in a separate breast-cancer surgery, without an NMBA; Nakamura et al. (2016) also reported uneventful rocuronium use during upper-limb orthopedic surgery [27,28]. In contrast, Johnson [6], Bouw [7], Tadokoro [11], and Nakamura [29] each reported clinical weakness following full TOF recovery. This dissociation between TOF recovery and clinical muscle strength may be explained by the predominantly central nature of postoperative weakness in SPS, where GABAergic over-suppression reduces respiratory drive rather than peripheral NMBA residual being the cause [7,8]. TOF monitoring can exclude peripheral NMBA residual but is insufficient as the sole criterion for extubation. Clinical assessments including sustained head lift [6], hand grip strength, tidal volume [7,11], and respiratory rhythm remain essential.

In this case, facial nerve monitoring requirements dictated NMBA avoidance. For clinical practice, the decision to use NMBAs should be driven by surgical necessity and disease severity. When muscle relaxation is required, rocuronium with quantitative TOF monitoring has been reported in SPS cases [10,18,30,31]; sugammadex was used for reversal in the three more recent reports [18,30,31]. When NMBA use can be avoided, as in this case where topical airway anesthesia and deep anesthesia facilitated intubation, avoidance removes one source of postoperative recovery uncertainty. This is particularly relevant for patients with greater disease severity or higher preoperative GABAergic drug loads.

Three additional considerations guided the anesthetic plan. First, TIVA with propofol, remifentanil, and dexmedetomidine was selected to avoid the potential interaction between volatile anesthetics and baclofen described in a prior SPS case [7]. TIVA and volatile anesthesia have both been used successfully in SPS; technique selection should be individualized according to clinical phenotype, medication burden, surgical requirements, and neuromonitoring needs [10,17,19,30]. For this patient, TIVA aligned with the prolonged microsurgical procedure and cranial nerve monitoring requirements. Second, dexmedetomidine provided a non-GABAergic adjunct to propofol. The earlier biopsy under dexmedetomidine MAC was well tolerated and informed the anesthetic plan. Third, remifentanil was maintained at 0.15 μg/kg/min with intermittent sufentanil boluses. BIS guidance (target 30–50) supported titration of anesthetic depth and has previously been used in SPS cases [16,32].

Regarding the extubation strategy, both Tadokoro et al. (2012) and Nakamura et al. (2007) encountered inadequate tidal volumes despite full TOF recovery after thymectomy, ultimately requiring overnight ventilation or ICU observation [11,29]. Those cases shared features including volatile anesthetic or NMBA use, thymoma-associated SPS, and bulbar involvement. Although volatile anesthetics and NMBAs were avoided in the present case, the combination of prolonged surgery, potential upper airway edema, and reports of respiratory insufficiency despite normalized TOF [7,11,29,31] supported planned delayed extubation. Recovery after 3.5 h in the ICU supports tailoring extubation timing to airway status, neuromuscular recovery, respiratory function, and surgical context. For shorter procedures, regional anesthesia or MAC sedation may be considered when compatible with surgical requirements [20]. Remimazolam with remifentanil has also been reported when general anesthesia was required [33].

### 3.2. Phenotype-Informed Perioperative Considerations

The four SPSD clinical types defined by Martinez-Hernandez et al. (2016) differ in antibody profiles, spasm severity, and prognosis [4]. SPSD includes focal stiff-limb phenotypes even when generalized spasms are not documented [4,23]. In a 2026 pooled review of 64 anti-amphiphysin SPSD cases, 37 of 45 patients with site-level data (82.2%) had lower-limb involvement; malignancy was present in 60 of 64 cases (93.8%), including breast cancer in 47 (73.4%) and lung cancer in 9 (14.1%) [25]. These findings support consideration of a paraneoplastic mechanism in anti-amphiphysin-positive SPSD [25]. In the literature identified for this revision, no PMT/TIO-associated anti-amphiphysin SPSD case was found. Under the 2021 PNS-Care criteria, an unusual cancer–antibody pairing requires demonstration of antigen expression by the tumor for a definite PNS diagnosis [34]. Evaluation of tumor amphiphysin expression, paired antibody titers, and long-term neurological response after resection would help clarify the PMT-SPSD relationship. Current evidence therefore supports two concurrent processes: PMT-associated TIO and anti-amphiphysin-positive, lower-limb-predominant SPSD. The specific PMT-SPSD relationship remains to be clarified through tumor-specific and longitudinal evidence. Perioperative decisions prioritized the documented clinical phenotype, medication burden, and surgical requirements.

Drawing on the SPSD classification [1,2,4] and case-based anesthesia literature [8,16,22,31], we propose a conceptual, hypothesis-generating framework to organize individualized perioperative planning and provide a basis for future evaluation (Table 1). SPS-plus/PERM or classic SPS with frequent airway-threatening spasms may warrant heightened precautions, including environmental control, adequate anesthetic depth, availability of NMBA reversal, and consideration of postoperative ICU monitoring. Classic SPS with occasional spasms but no respiratory involvement may represent an intermediate-concern phenotype, for which anesthetic technique should be individualized and known triggers avoided. For patients with stiff-limb presentations or minimal-to-no documented spasms, planning should emphasize surgical requirements and comorbidities while continuing to assess bulbar, respiratory, and medication-related risks. Together, these categories organize phenotype-related considerations for individualized planning.

Anesthetic technique should be matched to the clinical and surgical context. Regional anesthesia, including paravertebral block [20], epidural [9,15], and spinal anesthesia [14,35], may be considered when compatible with the procedure, although puncture-induced spasms and technical difficulty from spinal rigidity remain concerns [14]. For prolonged surgery in this patient, TIVA with dexmedetomidine, BIS guidance, and planned ICU observation were considered reasonable.

### 3.3. Limitations

As a single case, this report provides phenotype-specific perioperative experience. TOF monitoring data represent clinical time-point recordings rather than continuous waveform tracings. This report describes the inpatient course; longer-term neurological follow-up will be important to characterize subsequent SPS symptoms and functional recovery. The proposed framework provides a basis for evaluation in larger case series and systematically collected clinical evidence.

## 4. Conclusions

To our knowledge, this is the first published report of lateral skull base surgery in a patient with SPS and demonstrates that an 11-h muscle-relaxant-free TIVA protocol was feasible in one selected patient. The initial biopsy under dexmedetomidine MAC was well tolerated and informed the anesthetic plan. Perioperative management should be individualized according to the clinical phenotype, chronic medications, respiratory status, and surgical requirements. TIVA and volatile anesthesia have both been used successfully; technique selection should follow the clinical phenotype, surgical requirements, and neuromonitoring needs. NMBA use should reflect surgical necessity and disease severity, and TOF monitoring should be supplemented by clinical assessment before extubation. The phenotype-informed framework provides a structured approach to individualized perioperative planning and identifies priorities for future evaluation.

## Figures and Tables

**Table 1:** Proposed Hypothesis-Generating Phenotype-Informed Perioperative Framework.

Illustrative Category	SPSD Subtype	Clinical Features	Anesthetic Considerations
High	SPS-plus/PERM; classic SPS with frequent airway-threatening spasms	Frequent generalized spasms, autonomic instability	Consider stricter environmental control and adequate anesthetic depth; ensure NMBA reversal capability; consider postoperative ICU monitoring
Intermediate	Classic SPS (occasional spasms, no respiratory involvement)	Occasional painful spasms, startle sensitivity	Individualize anesthetic technique; avoid known triggers; use quantitative TOF monitoring if NMBAs are administered
Low	SLS; SPSD phenotype with minimal or no documented spasms	Predominantly focal or axial rigidity; no generalized spasms or current respiratory involvement	Base the anesthetic plan on surgical requirements and comorbidities; use NMBAs when indicated with appropriate monitoring

Note: This conceptual framework organizes phenotype-informed perioperative considerations and generates questions for future study. These categories indicate relative planning emphasis and organize clinical considerations. SPSD phenotypes exist along a continuum; final assessment integrates the individual history, current examination, medication burden, planned procedure, comorbidities, and local resources.

## Data Availability

The data supporting the central findings of this case report are contained within the article. Additional de-identified information may be available from the corresponding author upon reasonable request, subject to patient confidentiality and institutional requirements.

## Supplementary Materials

The following supporting information can be downloaded at: https://www.mdpi.com/article/10.3390/jcm15218217/s1, Checklist S1: Completed CARE checklist.
