# Supplementary material for: Anesthetic Management of Stiff-Person Syndrome During Prolonged Lateral Skull Base Surgery: A Case Report and Phenotype-Informed Review
Source: J Clin Med. 2026 Oct 22;15(21):8217. doi: 10.3390/jcm15218217 (PMC13763661; doi:10.3390/jcm15218217)
Supplement: Supplementary file 1 [file jcm-15-08217-s001.zip › jcm-4524644-supplementary.pdf]

CARE
case report guidelines

CARE Checklist of information to include when writing a case report

(cc) BY-NC-ND

Topic	Item	Checklist item description	Reported on Line
**Title**	1	The diagnosis or intervention of primary focus followed by the words "case report"	2-4
**Key Words**	2	2 to 5 key words that identify diagnoses or interventions in this case report, including "case report"	38-39
**Abstract (no references)**	3a	Introduction: What is unique about this case and what does it add to the scientific literature?	15-20
	3b	Main symptoms and/or important clinical findings	21-23
	3c	The main diagnoses, therapeutic interventions, and outcomes	21-31
	3d	Conclusion—What is the main "take-away" lesson(s) from this case?	32-37
**Introduction**	4	One or two paragraphs summarizing why this case is unique (**may include references**)	58-70
**Patient Information**	5a	De-identified patient specific information.	75-89
	5b	Primary concerns and symptoms of the patient.	75-79
	5c	Medical, family, and psycho-social history including relevant genetic information	75-89 (partial)*
	5d	Relevant past interventions with outcomes	84-98
**Clinical Findings**	6	Describe significant physical examination (PE) and important clinical findings.	78-108
**Timeline**	7	Historical and current information from this episode of care organized as a timeline	Figure 1; 115-116
**Diagnostic Assessment**	8a	Diagnostic testing (such as PE, laboratory testing, imaging, surveys).	78-108; 122-124
	8b	Diagnostic challenges (such as access to testing, financial, or cultural)	99-108; 234-241
	8c	Diagnosis (including other diagnoses considered)	80-108; 239-243
	8d	Prognosis (such as staging in oncology) where applicable	Not reported*
**Therapeutic Intervention**	9a	Types of therapeutic intervention (such as pharmacologic, surgical, preventive, self-care)	84-113; 118-147
	9b	Administration of therapeutic intervention (such as dosage, strength, duration)	118-147 (partial)*
	9c	Changes in therapeutic intervention (with rationale)	85-88; 140-147
**Follow-up and Outcomes**	10a	Clinician and patient-assessed outcomes (if available)	149-153 (clinician)*
	10b	Important follow-up diagnostic and other test results	151-153
	10c	Intervention adherence and tolerability (How was this assessed?)	95-96; 118-153*
	10d	Adverse and unanticipated events	136-139; 149-153
**Discussion**	11a	A scientific discussion of the strengths AND limitations associated with this case report	269-274; 276-286
	11b	Discussion of the relevant medical literature **with references**.	156-267
	11c	The scientific rationale for any conclusions (including assessment of possible causes)	170-243; 244-267
	11d	The primary "take-away" lessons of this case report (without references) in a one paragraph conclusion	276-286
**Patient Perspective**	12	The patient should share their perspective in one to two paragraphs on the treatment(s) they received	Not reported*
**Informed Consent**	13	Did the patient give informed consent? Please provide if requested	Yes ☑ No ☐

jcm-4524644 | * See completion notes on page 2. Written consent: manuscript lines 295-296.

CARE Checklist Completion Notes

Manuscript ID: jcm-4524644

Manuscript: Anesthetic Management of Stiff-Person Syndrome During Prolonged Lateral Skull Base Surgery: A Case Report and Phenotype-Informed Review

Line references correspond to the supplied clean manuscript.

5c: The medical history, medications and comorbidities are reported in lines 75-89. Family, psychosocial and relevant genetic history are not reported.

7: Figure 1 provides the clinical timeline; its caption is at lines 115-116. The clinical sequence is also described in Sections 2.1-2.3.

8b: Lines 99-108 and 234-241 discuss diagnostic overlap between tumor-induced osteomalacia and SPSD, and uncertainty regarding the PMT-SPSD relationship. Testing-access, financial or cultural barriers are not reported.

8d: A patient-specific long-term prognosis or tumor stage is not reported. Follow-up is limited to the available inpatient course (lines 149-153 and 269-274).

9b: Perioperative medication doses and timing are detailed in lines 118-147. The full doses and durations of earlier immunotherapy, phosphate/calcitriol/vitamin D therapy and postoperative patient-controlled analgesia are not reported.

10a: Clinician-assessed inpatient outcomes are reported in lines 149-153. A separate patient-assessed outcome measure is not reported.

10c: Tolerability of the initial biopsy and perioperative treatment is described (lines 95-96 and 118-153). A formal assessment of treatment adherence is not reported.

12: A patient-perspective narrative is not included in the supplied manuscript.

13: Written informed consent for publication and the accompanying clinical data is reported in lines 295-296.
